# Peroral endoscopic tumor resection of a large esophageal leiomyoma using refined traction and extraction techniques

**DOI:** 10.1055/a-2776-5290

**Published:** 2026-02-03

**Authors:** Yuto Shimamura, Marios Efthymiou, Vicki McGarrigle, Sujievvan Chandran, David S. Liu, Alex Craven, Rhys Vaughan

**Affiliations:** 1 3805 Department of Gastroenterology and Hepatology, Austin Health, University of Melbourne, Melbourne, Australia; 2 3805 Upper Gastrointestinal Surgery Unit, Division of Surgery, Anaesthesia and Procedural Medicine, Austin Health, Melbourne, Australia; 3 Victorian Interventional Research and Trials Unit, Department of Surgery, University of Melbourne, Melbourne, Australia; 4 3085 Division of Cancer Surgery, Peter MacCallum Cancer Centre, Melbourne, Australia

Peroral endoscopic tumor resection (POET) is a minimally invasive technique that enables the removal of subepithelial lesions (SELs) of the esophagus and stomach 1 2 . The optimal strategy for managing large esophageal SELs has not been standardized due to technical challenges 3 .

**Fig. 1:**
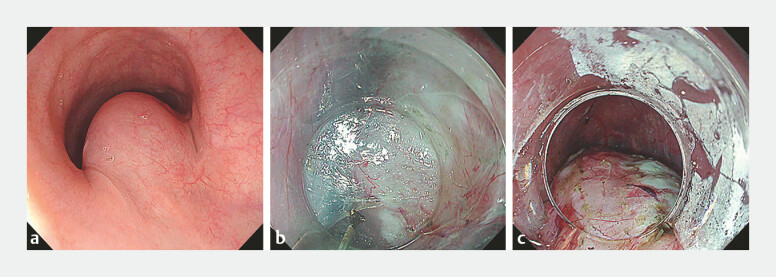
**a** A large esophageal subepithelial lesion measuring 5 cm in its longest (cranio-caudal) dimension and 2 cm in its minor axis, located in the proximal esophagus. **b** Submucosal tunneling and dissection were performed to enucleate the tumor. **c** The tumor was successfully enucleated from the mucosal side.

A 45-year-old woman was referred for POET of a large esophageal SEL measuring 5 cm in its longest (cranial-to-caudal) dimension and 2 cm in its minor axis ( Fig. 1 **a** ). Progressive growth had resulted in worsening dysphagia, prompting consideration of resection. Endoscopic ultrasound guided fine-needle biopsy confirmed the diagnosis of leiomyoma. Computed tomography (CT) revealed a mass extending from the T2–T4 vertebral levels, causing luminal narrowing. Following multidisciplinary discussion, POET was recommended, as surgical resection would have required both thoracic and cervical approaches and provided inadequate access to the full extent of the tumor due to its location and size.

**Fig. 2:**
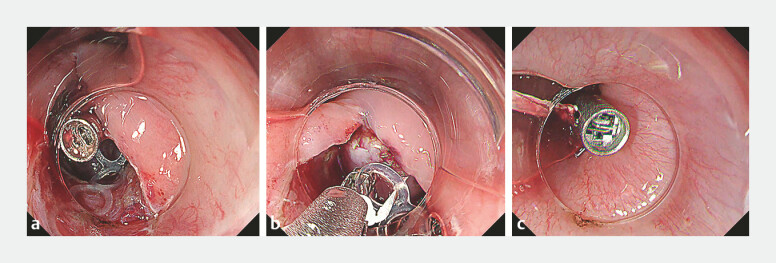
**a** A clip-and-band was deployed on the tumor surface. **b** A clip-and-line was attached to the band to provide traction with flexible direction. **c** Traction was applied from the oral side by pulling the line with the clip-and-line traction technique.

**Fig. 3:**
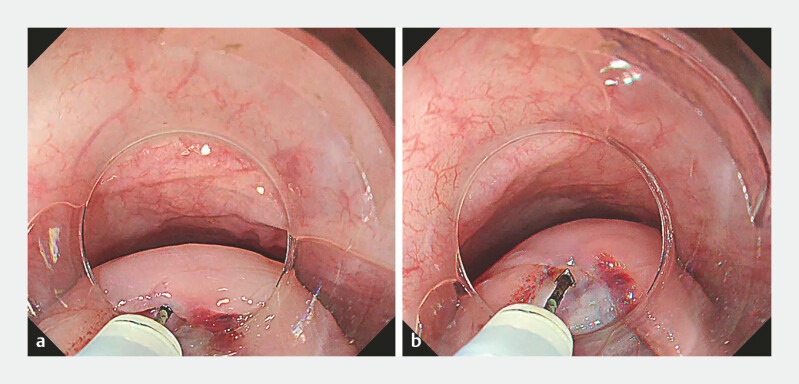
**a** An intentional mucosal incision was added to enhance visibility within the tunnel and facilitate smooth tumor extraction. **b** An additional 2 cm mucosal incision was made.

**Fig. 4:**
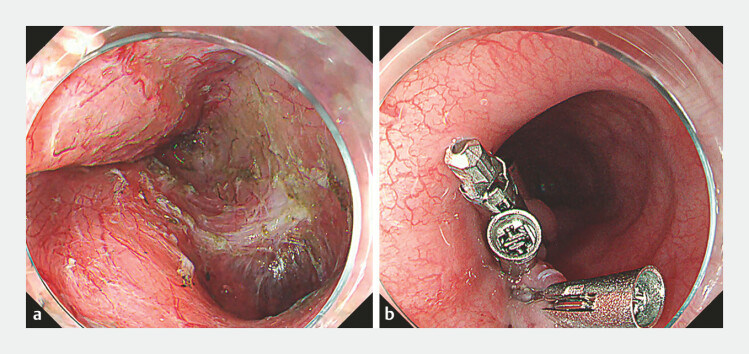
**a** Post-extraction showing the tumor originated from the muscular layer. **b** The mucosal incision site was closed with six clips.

POET was performed using a GIF-H290T gastroscope (Olympus, Tokyo, Japan) and a Triangle Tip-Jet knife (KD-645L; Olympus). A 2-cm mucosal incision was made approximately 2 cm proximal to the tumor at 18 cm from the incisor, and a submucosal tunnel was created and extended beyond the distal margin of the lesion. The tumor was meticulously dissected from the submucosal and muscular layers ( Fig. 1 **b, c** ). Due to the tumor’s size, two technical modifications were applied: (1) a clip-and-band combined with the clip-with-line method was used to optimize visualization within the tunnel by pulling the tumor outward ( Fig. 2 **a–c** ), and (2) a 2-cm extended mucosal incision was made to facilitate extraction ( Fig. 3 **a–b** ). Once fully enucleated, it was extracted using a snare, and the entry site was closed with six clips ( Fig. 4 **a, b** ). En bloc resection was achieved without adverse events, and the patient was discharged the following day.

This case demonstrates that POET with traction-assisted visualization and extended mucosal incision can be safely and effectively used for large esophageal subepithelial lesions ( Video 1 ).

Peroral endoscopic tumor resection (POET) of a large esophageal leiomyoma located in the proximal esophagus. Source for the histological and immunohistochemical images: Rhoda Cameron, Austin Health.Video 1

Endoscopy_UCTN_Code_TTT_1AO_2AG_3AF
